# Nationwide prospective survey of secondary myelofibrosis in Japan: superiority of DIPSS-plus to MYSEC-PM as a survival risk model

**DOI:** 10.1038/s41408-023-00869-9

**Published:** 2023-07-19

**Authors:** Kotaro Shide, Katsuto Takenaka, Akira Kitanaka, Akihiko Numata, Takuro Kameda, Takuji Yamauchi, Atsushi Inagaki, Shohei Mizuno, Akiyoshi Takami, Shinichi Ito, Masao Hagihara, Kensuke Usuki, Takaaki Maekawa, Kazutaka Sunami, Yasunori Ueda, Miyuki Tsutsui, Miki Ando, Norio Komatsu, Keiya Ozawa, Mineo Kurokawa, Shunya Arai, Kinuko Mitani, Koichi Akashi, Kazuya Shimoda

**Affiliations:** 1 Division of Hematology, Diabetes, and Endocrinology, Department of Internal Medicine, Faculty of Medicine, University of Miyazaki, 5200 Kihara Kiyotake, Miyazaki, 889-1692 Japan; 2 Department of Hematology, Clinical Immunology, and Infectious Diseases, Ehime University Graduate School of Medicine, Touon-shi, Ehime Japan; 3 Department of Laboratory Medicine, Kawasaki Medical School, Kurashiki, Japan; 4 Department of Medicine and Biosystemic Science, Kyushu University Graduate School of Medical Science, Fukuoka, Japan; 5 Department of Hematology and Oncology, Nagoya City West Medical Center, Nagoya, Japan; 6 Division of Hematology, Department of Internal Medicine, Aichi Medical University School of Medicine, Nagakute, Japan; 7 Department of Hematology, Hakodate City Hospital, Hakodate, Japan; 8 Department of Hematology, Eiju General Hospital, Tokyo, Japan; 9 Department of Hematology, NTT Medical Center Tokyo, Tokyo, Japan; 10 Division of Hematology, Department of Internal Medicine, National Defense Medical College, Tokorozawa, Japan; 11 Department of Hematology, National Hospital Organization Okayama Medical Center, Okayama, Japan; 12 Department of Hematology/Oncology, Kurashiki Central Hospital, Okayama, Japan; 13 Department of Hematology, Juntendo University School of Medicine, Tokyo, Japan; 14 Division of Hematology, Department of Medicine, Jichi Medical University, Tochigi, Japan; 15 Department of Hematology and Oncology, Graduate School of Medicine, The University of Tokyo, Tokyo, Japan; 16 Department of Hematology and Oncology, Dokkyo Medical University, Tochigi, Japan

**Keywords:** Myeloproliferative disease, Cancer epidemiology, Risk factors

Dear editor,

Myelofibrosis (MF) is divided into two categories, namely primary MF (PMF) and secondary MF arising from essential thrombocythemia (ET) or polycythemia vera (PV), so-called PET-MF or PPV-MF, respectively [1]. ET and PV are considered dormant MPNs, and their progression to MF is a natural evolution. 15 years-cumulative incidence of PET-MF and PPV-MF were 4–11% and 6–14%, respectively [2]. Patients with PET/PPV-MF and PMF are similarly managed in clinical practice [3]. However, discrepancies in the application of PMF prognostic scoring systems, such as the International Prognostic Scoring System (IPSS) and dynamic IPSS (DIPSS) for patients with PET/PPV-MF have been reported [4–8], and the prognostic scoring systems for PET/PPV-MF has been developed [9]. We evaluated the existing prognostic models developed for PMF and PET/PPV-MF in a nationwide prospective observational study for patients with PET/PPV-MF conducted by the Japanese National Research Group on Idiopathic Bone Marrow Failure Syndromes.

A total of 272 patients diagnosed with PET-MF (*n* = 163) or PPV-MF (*n* = 109) from 2012 to 2020 were included (Fig. S1). PET/PPV-MF was diagnosed by the International Working Group on Myelofibrosis Research and Treatment (IWG-MRT) criteria [10]. Karyotype classification followed the previously reported classification [7]. This study was approved by the Research Ethics Committee of the University of Miyazaki, and those of other participating institutes in accordance with the Helsinki Declaration. The Kaplan–Meier method was used to estimate overall survival (OS), and the log-rank test was used to assess differences in OS among the patient groups. The effects of risk factors on OS were evaluated by Cox proportional hazards regression modeling. The predictions were evaluated using the hazard ratio (HR), log-rank test, and concordance index (C-index) [11].

The clinical characteristics of the patients are summarized in Table S1. The median age at diagnosis was 70.0 years, 52.0% were males, and the median time from ET or PV to MF diagnosis was 10.1 years. The type and frequency of driver mutations reflected the primary disease. PET-MF displayed *JAK2*V617F in 57.4% (81/141), *CALR* in 23.7% (28/118), *MPL* in 4.2% (5/118), and triple-negative in 3.4% (4/118). PPV-MF displayed *JAK2*V617F in 94.9% (93/98). Cytogenetic data was obtained in 80.1% (218/272) of the patients using bone marrow cells or peripheral blood leukocytes, with 56.4% (123/218) showing a normal karyotype and 43.6% (95/218) showing some type of chromosomal abnormality. Unfavorable karyotypes were observed for 17.0% (37/218) [7]. As for treatments, ruxolitinib (RUX) was the most common drug therapy (78.3%, *n* = 213), followed by hydroxyurea (60.3%, *n* = 164). Twenty (7.4%) patients underwent allogeneic hematopoietic cell transplantation (HCT) (Table S1).

The median follow-up time was 2.47 years (range, 0–9.67). The 3-year OS was 0.73, and the median survival time was 6.33 years (Fig. S2A). Seventy-seven patients died during the observation period. The main causes of death were leukemic transformation (42%, *n* = 32) and infections (23%, *n* = 18). We determined if the known prognostic factors used in the IPSS, DIPSS, DIPSS-plus, and the myelofibrosis secondary to PV and ET prognostic model (MYSEC-PM) were predictive of shortened survival in patients with PET/PPV-MF (Table S2). The five factors included in the IPSS/DIPSS had independent predictive values. However, an 8-factor multivariate analysis of the DIPSS-plus with three factors (Plt < 100 × 10^9^/L, transfusion dependence, and unfavorable karyotype) showed that only three factors: WBC > 25 × 10^9^/L, transfusion dependence, and unfavorable karyotype, had independent predictive value. This suggests that these three factors have a stronger impact on survival than other factors. Regarding the prognostic factors included in the MYSEC-PM, four factors, excluding constitutional symptoms and *CALR* unmutated genotype, were independently associated with shortened survival according to multivariate analysis. Kaplan–Meier curves of karyotype and mutation genotype are shown in Fig. S2B. A significant survival stratification was observed when patients were divided into two groups, unfavorable and all other karyotypes. On the other hand, no significant difference in survival between patients with *CALR* mutated and *CALR* unmutated genotypes was observed (*p* = 0.45). The impact of chromosomal abnormalities on shortened survival is equally large in secondary MF, as previously reported for PMF [7].

We evaluated the performance of IPSS, DIPSS, DIPSS-plus, and MYSEC-PM. IPSS and DIPSS were applicable in all 272 cases. Depending on the available information on karyotype or driver mutation genotype, the DIPSS-plus or MYSEC-PM was applied in 218 and 224 patients, respectively (Fig. S1). There were 183 patients for whom all four models were applicable. The results of applying each model to the maximum number of cases are shown in Fig. 1A. Table 1 summarizes the predicted 3-year survival rate for the patients in each risk category and the HR of the adjacent lower-risk category. The int-1 and int-2 risk groups were differentiated based on the HRs in all four models. The IPSS, DIPSS, and DIPSS-plus models also significantly differentiated the int-2 and high-risk groups, whereas the MYSEC-PM failed to differentiate the high-risk and int-2 risk groups (HR, 1.78; 95% CI: 0.95–3.33, *p* = 0.081). The overall predictive performance of the models was evaluated using Harrell’s C-statistics (Fig. 1A). The DIPSS-plus had the highest C-index (0.887 (95% CI: 0.830–0.943)), followed by IPSS and DIPSS. Meanwhile, the MYSEC-PM had the lowest C-index (0.806 (95% CI: 0.736–0.877)). The analysis of a common cohort of 183 cases showed similar results (Fig. S3). The risk classification of each patient for DIPSS-plus and MYSEC-PM was visualized (Fig. 1B). There were 33 and 43 patients in the high-risk group by DIPSS-plus and MYSEC-PM, respectively (indicated by red bars). Of these, 11, 21, and 22 were classified as high risk by DIPSS-plus only, MYSEC-PM only, and both DIPSS-plus and MYSEC-PM, respectively. The survival rates of patients in these three groups are shown in Fig. 1C. Patients in the high-risk group on both DIPSS-plus and MYSEC-PM did have a poor prognosis. High-risk patients with DIPSS-plus alone had an equally poor prognosis, whereas high-risk patients with MYSEC-PM alone had a better prognosis. These data indicate that the DIPSS-plus could identify cases with poor prognoses more effectively than the MYSEC-PM.

**Fig. 1:**
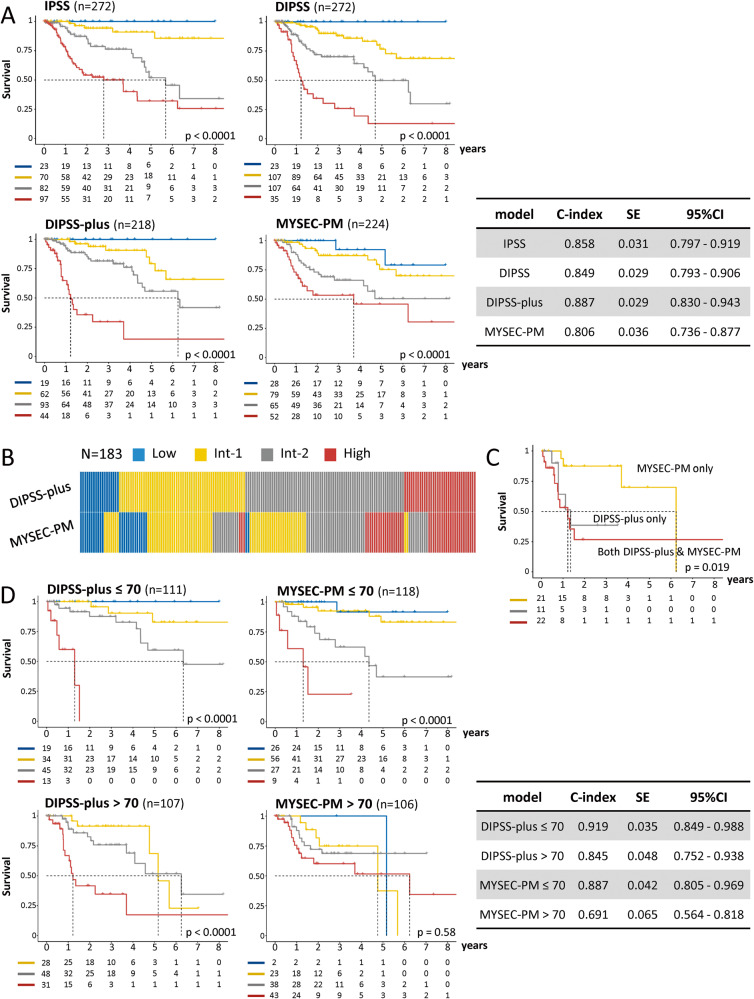
Application of current prognostic models in patients with PET/PPV-MF. **A** IPSS, DIPSS, DIPSS-plus, and MYSEC-PM were applied to the maximum number of cases for each model. The C-index for each model is shown. The p-values from the log-rank test are shown in the figure. **B** Concordance chart of patients classified with the DIPSS-plus and the MYSEC-PM. A vertical column represents a single patient. **C** Kaplan–Meier curves for cases classified as high-risk by the DIPSS-plus or MYSEC-PM. Patients in the high-risk group on both DIPSS-plus and MYSEC-PM had poor prognoses (red line). High-risk patients with DIPSS-plus alone had an equally poor prognosis (gray line), whereas high-risk patients based on the MYSEC-PM alone had a better prognosis (yellow line). The *p*-values from the log-rank test are shown in the figure. **D** Application of DIPSS-plus and MYSEC-PM to a cohort divided into two based on age 70. The C-index for each model is shown. The *p*-values from the log-rank test are shown in the figure.

**Table 1:** Projected survival according to the risk categories by the prognostic scoring system model when each was applied to the maximum available cases.

Risk category	*n* (%)	3-year OS	HR (95% CI)	*P*
***IPSS***	*n* = 272			
Low	23 (8.5)	1.0		
			NA	0.211
Int-1	70 (25.7)	0.943		
			4.97 (1.86–13.28)	**0.001**
Int-2	82 (30.1)	0.762		
			2.31 (1.36–3.93)	**0.002**
High	97 (35.7)	0.500		
***DIPSS***	*n* = 272			
Low	23 (8.5)	1.0		
			NA	0.071
Int-1	107 (39.3)	0.881		
			3.00 (1.60–5.61)	**<0.001**
Int-2	107 (39.3)	0.701		
			2.98 (1.71–5.17)	**<0.001**
High	35 (12.9)	0.259		
***DIPSS-plus***	*n* = 218			
Low	19 (8.7)	1.0		
			NA	0.141
Int-1	62 (28.4)	0.906		
			2.38 (1.06–5.36)	**0.036**
Int-2	93 (42.7)	0.815		
			4.95 (2.62–9.34)	**<0.001**
High	44 (20.2)	0.297		
***MYSEC-PM***	*n* = 224			
Low	28 (12.5)	0.923		
			2.06 (0.46–9.31)	0.337
Int-1	79 (35.3)	0.872		
			2.71 (1.30–5.66)	**0.011**
Int-2	65 (29.0)	0.656		
			1.78 (0.95–3.33)	0.081
High	52 (23.2)	0.533		

*IPSS* International Prognosis Scoring System, *DIPSS* dynamic IPSS, *MYSEC-PM* myelofibrosis secondary to PV and ET prognostic model, *int-1* intermediate-1, *int-2* intermediate-2, *OS* overall survival, *HR* hazard ratio.

Bold values indicates statistically significant *P* values (*P* < 0.05).

The median age of the patients in the original MYSEC cohort was 64 years [9], while that in this study was 70 years. Therefore, we investigated whether the predictive power of the models differed by age (Fig. 1D). The MYSEC-PM functioned effectively in patients aged ≤70 years (C-index: 0.887), whereas its predictive power was significantly reduced in patients aged >70 years (C-index, 0.691). On the other hand, the DIPSS-plus exhibited superior classification of patients with PET/PPV-MF into different risk categories regardless of if they are aged ≤70 years or >70 years. The IPSS or DIPSS showed similar tendencies (Figure S4). In addition to age, the cut-off value of blasts percentage and Hb differed between DIPSS-plus and MYSEC-PM. However, when the cut-off value of blast ≥1% and/or that of Hb <10 g/dl were adopted in MYSEC-PM, The C-index (0.810–0.812) after changing the cutoff value was similar to that before changing (0.806).

Two studies have evaluated the prognostic models of PMF or MYSEC-PM in patients with PET/PPV-MF at diagnosis [5, 12]. Tefferi et al. applied the IPSS and DIPSS-plus in 125 cases of PET/PPV-MF (PET, 46. PPV, 79) at a single institution in Mayo. These models could not differentiate between the int-1- and int-2-risk groups; however, they could effectively differentiate between the int-2- and high-risk groups [5]. Boluda et al. applied IPSS and MYSEC-PM in 262 PET/PPV-MF cases (PET, 141; PPV, 121) from the Spanish registry. The IPSS could effectively differentiate between the int-1- and int-2-risk groups but could not separate between the int-2- and high-risk groups (HR, 1.4; 95% CI: 0.9–2.3, *p* = 0.12). On the other hand, the MYSEC-PM could differentiate all groups [12]. In our study, all PMF prognostic models, including the IPSS could effectively differentiate between the int-1- and int-2-risk groups and the int-2- and high-risk groups. However, the MYSEC-PM could not significantly differentiate between the int-2- and high-risk groups. The DIPSS-plus and MYSEC-PM had the highest and lowest C-index, respectively. The superiority of the DIPSS-plus over other models may be due to the greater impact of karyotype on prognosis. We considered the patients’ age at diagnosis as a reason why the MYSEC-PM was less effective in our study. The original MYSEC cohort comprised 685 patients with a median age of 64 years and 53% of the patients were aged >65 years. In contrast, the median age in our study was 70 years with 64% of the patients aged >65 years which was the highest compared to previous reports [5, 9, 12–14]. After dividing the cohort into those ≤70 and those >70 years, we found that the MYSEC-PM functioned effectively at ≤70 years of age (Fig. 1D). As a result of the higher scores assigned to age, the age distribution of patients with int-2- and high-risk according to the MYSEC-PM was skewed towards older ages compared to the IPSS or DIPSS-plus (Fig. S5). The excessive contribution of age to risk score may result in decreased prognostic accuracy for higher-risk groups.

In conclusion, the prognostic models for PMF, such as the DIPSS-plus, which contain karyotype with a strong impact on survival, may be useful for PET/PPV-MF patients in identifying HCT candidates and predicting the prognosis of patients even in the MYSEC-PM era.

## Supplementary information

Supplementary Information

## Data Availability

All data presented in the text are accompanied by relevant tables or figures in the “Results” section or Supplementary Materials. No individual patient data is shared.
