# Supplementary material for: Nationwide prospective survey of secondary myelofibrosis in Japan: superiority of DIPSS-plus to MYSEC-PM as a survival risk model
Source: Blood Cancer J. 2023 Jul 19;13(1):110. doi: 10.1038/s41408-023-00869-9 (PMC10354019; doi:10.1038/s41408-023-00869-9)

**Supplementary Information**

**Supplementary methods**

**Patients**

We conducted a nationwide prospective survey on secondary MF from 2012 in Japan. Annual questionnaires containing detailed clinical data were sent every year to the hematology departments of board-certified institutes of the Japanese Society of Hematology and were collected for the enrollment of patients with new-onset of secondary MF. The enrolled patients were followed up every year. From 2012 to 2020, 275 patients diagnosed with PET-MF (n=163) or PPV-MF (n=112) were registered, of which 272 were included in this study after excluding three patients with PPV-MF owing to insufficient information (**Figure S1**).

**Statistical analysis**

Patient characteristics were compared among various groups using the Wilcoxon rank sum test or one-way non-parametric ANOVA (Kruskal–Wallis test) for continuous variables and Fisher’s exact test or Chi-square test for categorical variables. The Holm procedure was used to adjust for multiple testing. P values were calculated using two-sided tests, where p < 0.05 was considered statistically significant. Statistical analyses were performed using R (version 4.1.2) and the R packages: survival (version 3.2-13) and survcomp (version 1.40.0).

**Table S1. Demographics and clinical features of patients at the diagnosis of PET-MF and PPV-MF**

|  | **Overall** |
| --- | --- |
| N | 272 |
| **Characteristics** |  |
| Age, years; median [IQR] | 70.0 [62.0, 76.0] |
| Age >65 years, n (%) | 173 (63.6) |
| Males, n (%) | 140 (52.0) |
| Time from ET/PV diagnosis to MF diagnosis, years; median [IQR] | 10.1 [5.5, 16.0] |
| Constitutional symptoms, n (%) | 89 (32.7) |
| Body weight loss, n (%) | 59 (23.0) |
| Fever, n (%) | 31 (11.8) |
| Night sweat, n (%) | 36 (13.9) |
| Splenomegaly, n (%) | 193 (71.0) |
| WBC, x10^9^/L, median [IQR] | 10.96 [6.30, 18.8] |
| WBC >25x10^9^/L, n (%) | 46 (16.9) |
| Hb, g/L, median [IQR] | 9.8 [8.2, 12.0] |
| Hb <10g/dL, n (%) | 141 (51.8) |
| Transfusion dependence, n (%) | 110 (40.4) |
| Plt, x10^9^/L, median [IQR] | 332 [195, 523] |
| Plt <100x10^9^/L, n (%) | 27 (9.9) |
| Circulating blast ≧1%, n (%) | 117 (43.0) |
| LDH, IU/L, median [IQR] | 498 [353, 718] |
| Abnormal karyotypes, n (%) | 95 (43.6) |
| Unfavorable karyotypes, n (%) | 37 (17.0) |
| **Treatment** |  |
| Any treatment, n (%) | 243 (89.3) |
| Ruxolitinib, n (%) | 213 (78.3) |
| Hydroxyurea, n (%) | 164 (60.3) |
| Prednisolone, n (%) | 23 (8.5) |
| Anabolic steroid, n (%) | 21 (7.7) |
| Azacytidine, n (%) | 15 (5.5) |
| Other (oral cytarabine, thalidomide), n (%) | 18 (6.6) |
| Splenic irradiation, n (%) | 6 (2.7) |
| Splenectomy, n (%) | 2 (0.8) |
| HCT, n (%) | 20 (7.4) |

Abbreviations: IQR, Interquartile Range; WBC, white blood cell count; Hb, hemoglobin; Plt, platelet; LDH, lactate dehydrogenase; HCT, Hematopoietic stem cell transplantation. Unfavorable karyotype: a complex karyotype or abnormalities including +8, -7/7q-, i(17q), -5/5q-, 12p-, inv(3) or 11q23 rearrangement.

**Table S2. Application of the current prognostic models: analysis of risk factors**

| **Variable** | **Univariate** | |  | **Multivariate** | |
| --- | --- | --- | --- | --- | --- |
|  | **HR (95% CI)** | **p** |  | **HR (95% CI)** | **p** |
| **IPSS/DIPSS risk factors (N=272)** | |  |  |  |  |
| Age > 65 years | 2.44 (1.37 - 4.36) | **0.002** |  | 2.42 (1.34 - 4.39) | **0.004** |
| Constitutional symptoms | 2.14 (1.32 - 3.46) | **0.002** |  | 1.67 (1.02 - 2.74) | **0.042** |
| Hb < 10 g/L | 4.27 (2.43 - 7.49) | **< 0.001** |  | 3.02 (1.67 - 5.46) | **< 0.001** |
| WBC >25 x 10^9^/L | 1.78 (1.00 - 3.16) | **0.050** |  | 2.47 (1.36 - 4.48) | **0.003** |
| Circulating blast ≧ 1% | 3.73 (2.23 - 6.26) | **< 0.001** |  | 2.61 (1.51 - 4.51) | **0.001** |
| **DIPSS-plus risk factors (N=218)** | |  |  |  |  |
| Age > 65 years | 2.57 (1.31 - 5.04) | **0.006** |  | 1.64 (0.79 - 3.42) | 0.186 |
| Constitutional symptoms | 2.25 (1.29 - 3.92) | **0.004** |  | 1.58 (0.89 - 2.82) | 0.117 |
| Hb < 10 g/L | 3.80 (2.05 - 7.04) | **< 0.001** |  | 1.82 (0.88 - 3.75) | 0.105 |
| WBC >25 x 10^9^/L | 1.84 (0.96 - 3.53) | 0.064 |  | 3.03 (1.51 - 6.09) | **0.002** |
| Circulating blast ≧ 1% | 3.43 (1.92 - 6.15) | **< 0.001** |  | 1.91 (0.99 - 3.69) | 0.053 |
| Plt < 100 x 10^9^/L | 3.69 (1.78 - 7.67) | **< 0.001** |  | 1.75 (0.79 - 3.84) | 0.166 |
| Transfusion dependency | 5.04 (2.84 - 8.95) | **< 0.001** |  | 2.67 (1.25 - 5.68) | **0.011** |
| Unfavorable karyotype | 1.96 (1.39 - 2.76) | **< 0.001** |  | 1.62 (1.07 - 2.46) | **0.024** |
| **MYSEC-PM risk factors (N=224)** | |  |  |  |  |
| Age | 1.05 (1.02 - 1.08) | **0.001** |  | 1.04 (1.01 - 1.07) | **0.007** |
| Constitutional symptoms | 1.97 (1.14 - 3.39) | **0.014** |  | 1.44 (0.82 - 2.54) | 0.204 |
| Hb < 11 g/L | 3.62 (1.86 - 7.03) | **< 0.001** |  | 2.60 (1.30 - 5.17) | **0.007** |
| Circulating blast ≧ 3% | 2.71 (1.47 - 5.01) | **0.001** |  | 2.05 (1.08 - 3.90) | **0.029** |
| Plt < 150 x 10^9^/L | 2.53 (1.37 - 4.69) | **0.003** |  | 2.06 (1.10 - 3.88) | **0.025** |
| *CALR* unmutated genotype | 1.43 (0.57 - 3.60) | 0.444 |  |  |  |

Abbreviations: Hb, hemoglobin; WBC, white blood cell count; Plt, platelet; LDH, lactate dehydrogenase; IPSS, International Prognosis Scoring System; DIPSS, Dynamic IPSS; MYSEC-PM, Myelofibrosis Secondary to PV and ET Prognostic Model; HR, hazard ratio. Unfavorable karyotypes: a complex karyotype or abnormalities including +8, -7/7q-, i(17q), -5/5q-, 12p-, inv(3) or 11q23 rearrangement.

**Supplementary Figure legends**

**Figure S1. Analysis flow of this research.**

After excluding three patients with post-polycythemia vera myelofibrosis (PPV-MF) due to insufficient information from 275 registered cases, 272 cases were included in this study. We evaluated the performance of the International Prognostic Scoring System (IPSS), Dynamic IPSS (DIPSS), DIPSS-plus, and the MF Secondary to PV and essential thrombocythemia (ET) Prognostic Model (MYSEC-PM) risk classifications of patients with PET/PPV-MF. The IPSS and DIPSS were applicable in all the 272 cases. The DIPSS-plus was applicable in 218 cases and MYSEC-PM, in 224 cases, depending on the presence of karyotype or driver mutation genotype information. There were 183 patients for whom all four models were applicable.

**Figure S2. Survival outcomes and causes of death.**

**(A)** Kaplan–Meier curve of all the 272 cases. The 3-year OS was 0.73 and median survival was 6.33 years.

**(B)** Survival according to karyotype and driver mutation status. **(left)** Survival according to the two categories of karyotypes. Unfavorable karyotypes include a complex karyotype or abnormalities including +8, -7/7q-, i(17q), -5/5q-, 12p-, inv(3), or 11q23 rearrangement. The p-values from the log-rank test are shown in the figure. **(right)** Survival with and without *CALR* mutation. *CALR* unmutated genotype includes *JAK2*V617F positive PPV-MF, *JAK2*V617F positive PET-MF, *MPL* mutated PET-MF, and triple-negative PET-MF patients.

**Figure S3. Application of the four current prognostic scoring systems for the common 183 cases.**

**(A)** Kaplan–Meier curve of the common 183 cases. The 3-year OS was 0.766 and median survival was not reached.

**(B)** Application of the four prognostic models to the common cases. The IPSS, DIPSS, and DIPSS-plus were able to significantly differentiate int-2 and high-risk groups, on the other hand the MYSEC-PM failed to differentiate these higher risk categories (HR, 1.76; 95% CI: 0.85–3.62, p=0.145). The DIPSS-plus had the highest C-index (0.885), followed by IPSS (0.852), DIPSS (0.843), and MYSEC-PM (0.799). The p-values from the log-rank test are shown in the figure.

**Figure S4. Application of the IPSS and DIPSS to a cohort grouped based on age 70.**

In patients aged ≤70 years, the IPSS and DIPSS could significantly differentiate int-1 and int-2 risk groups and int-2 and high-risk groups. In patients aged >70 years, these models failed to differentiate the int-1 and int-2 risk groups; however, it significantly differentiated the int-2 and high-risk groups. The C-index for each model is shown below. The p-values from the log-rank test are shown in the figure.

**Figure S5. Age distribution of the patients in the Int-2 and high categories by prognostic models.**

The box represents the median, and 25th and 75th percentiles. The dotted line indicates 70 years old. The p-values are shown below. The age distribution of the int-2/high category in the MYSEC-PM model was significantly higher than the IPSS or DIPSS-plus (both p=0.010).

Figure S1.


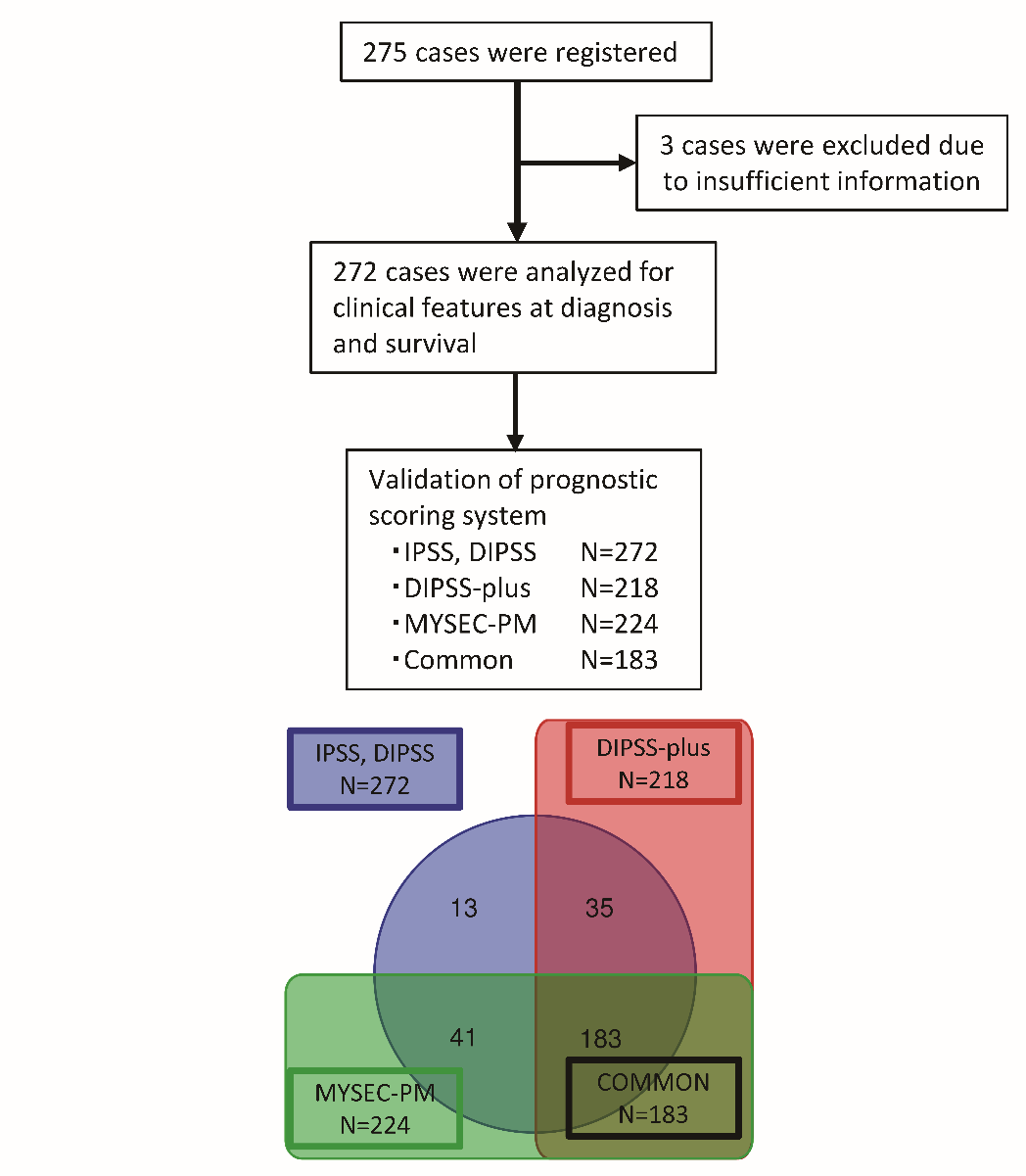


Figure S2.


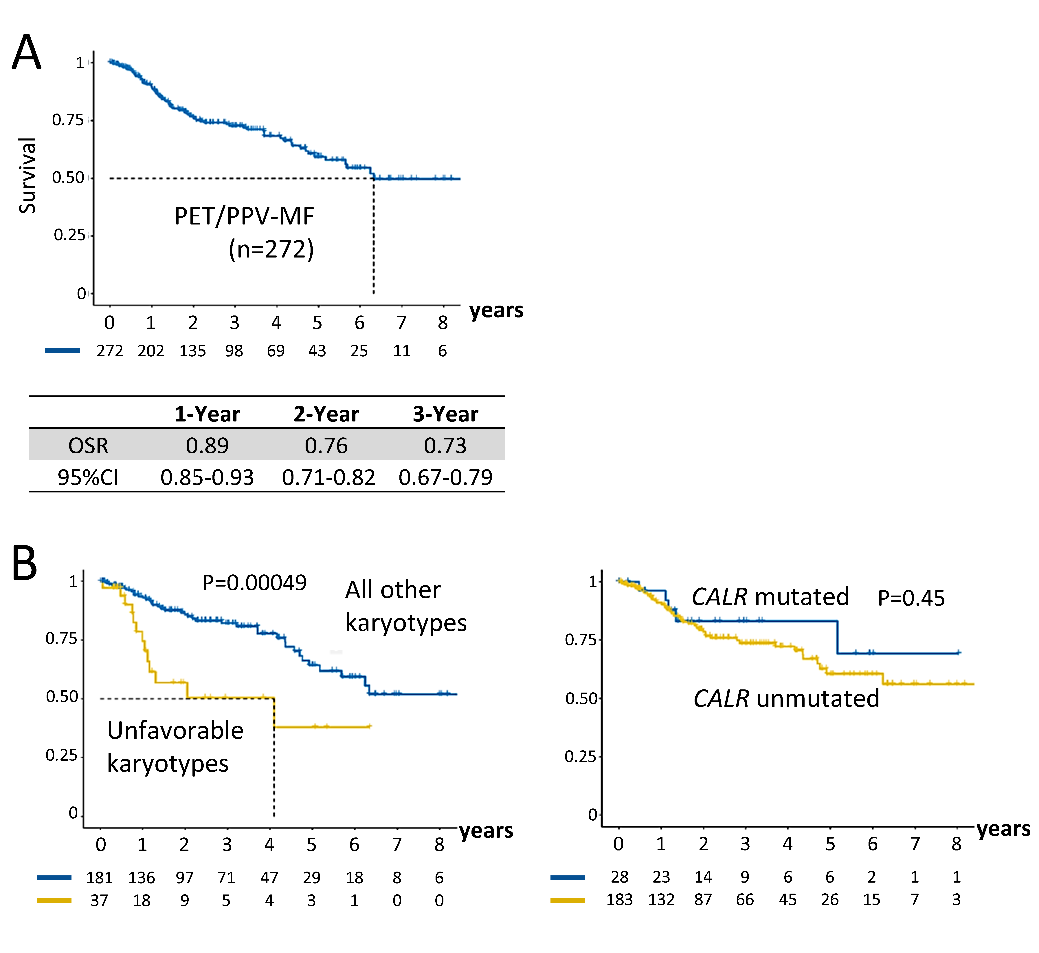


Figure S3.

**
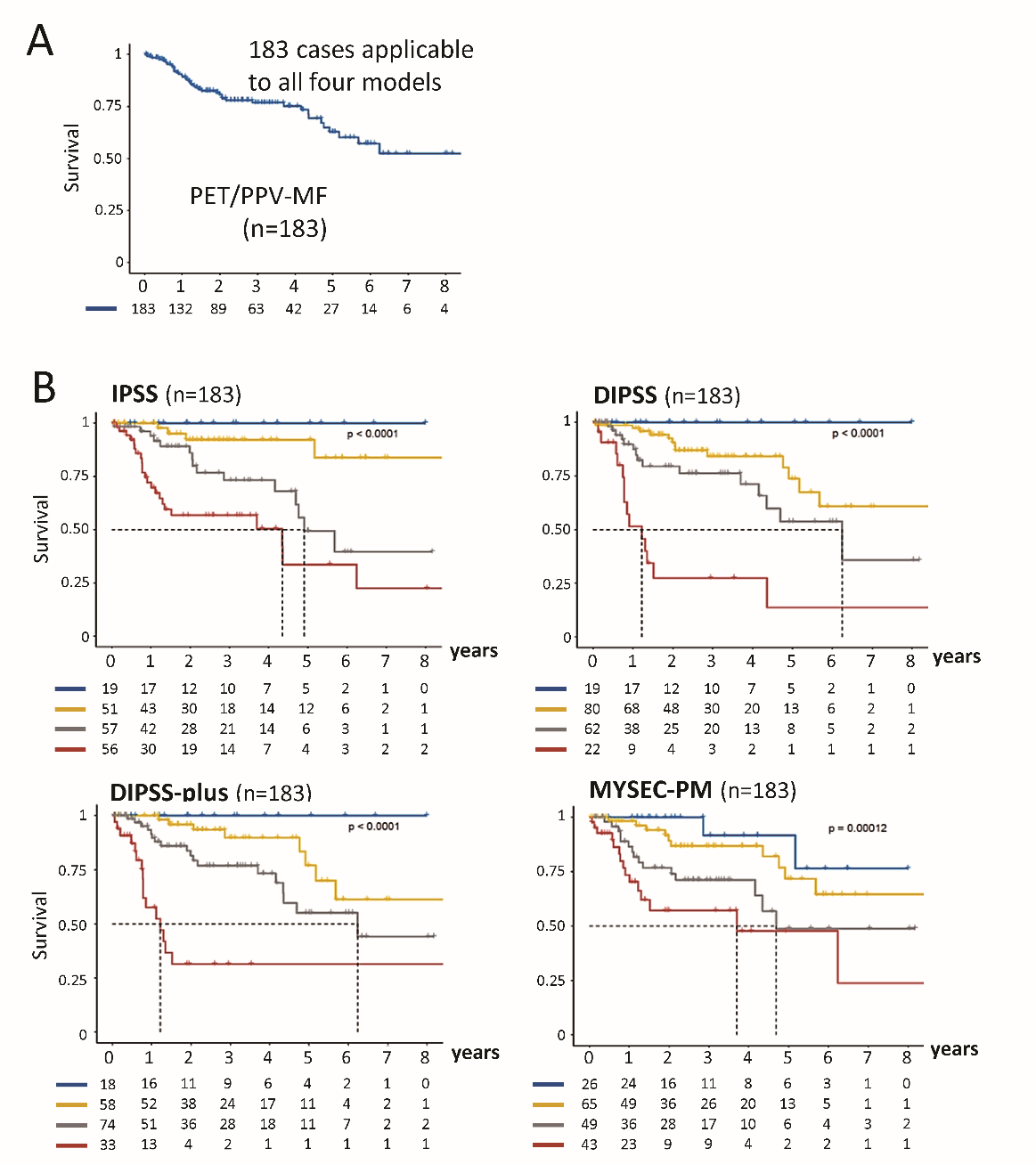
**

**
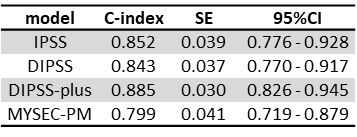
**

Figure S4.


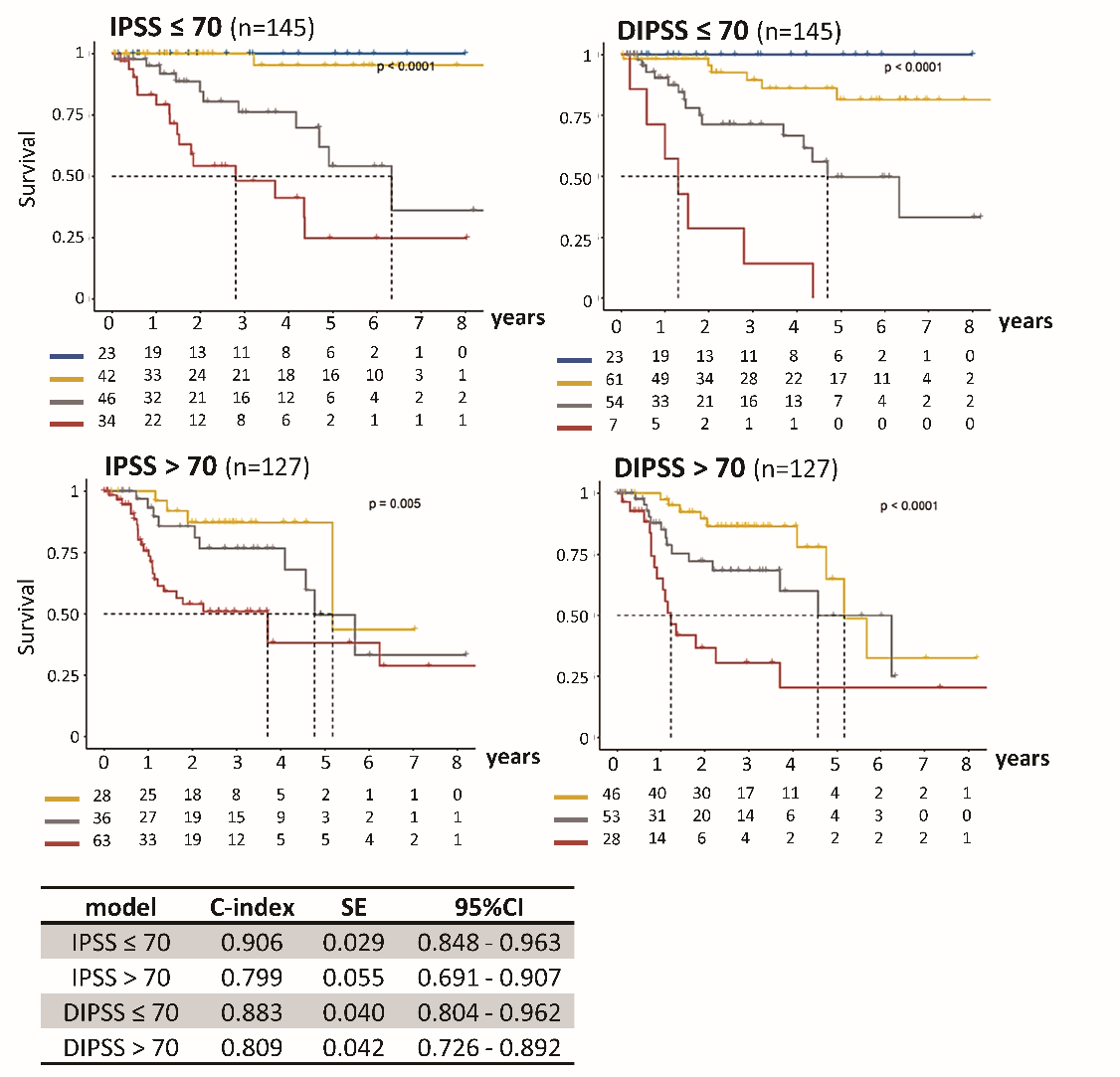


Figure S5.


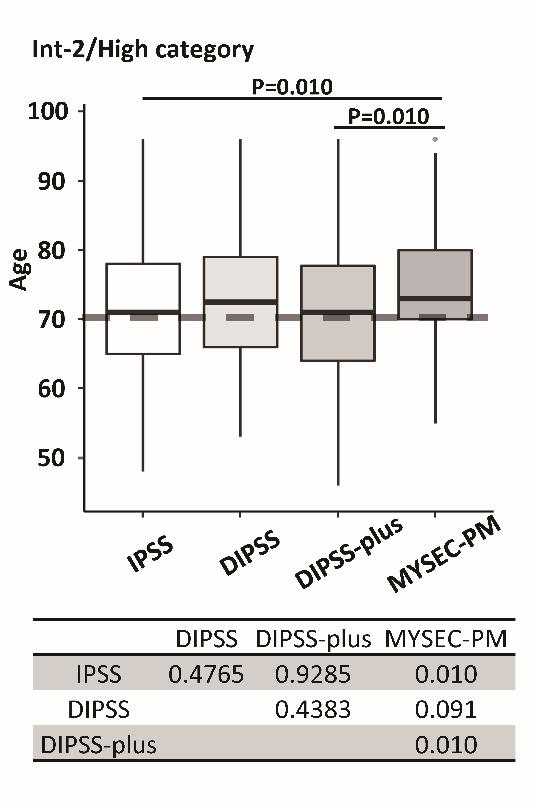

Supplement: Supplementary file 1 — Supplementary Information [file 41408_2023_869_MOESM1_ESM.docx]
